# Comprehensive analysis of the prognostic and immunological signature of eight Tripartitemotif (TRIM) family molecules in human gliomas

**DOI:** 10.18632/aging.204841

**Published:** 2023-06-24

**Authors:** Jiajie Lu, Kairong Liang, Renheng Zou, Yuecheng Peng, Haojian Wang, Rihong Huang, Zhaorong Zeng, Zejia Feng, Yongyang Fan, Shizhen Zhang, Yunxiang Ji, Xiao Pang, Yezhong Wang, Hongri Zhang, Zhaotao Wang

**Affiliations:** 1 Institute of Neuroscience, Department of Neurosurgery, The Second Affiliated Hospital of Guangzhou Medical University, Guangzhou 510260, China; 2 Department of Clinical Medicine, The Second Clinical School of Guangzhou Medical University, Guangzhou 510182, China; 3 Department of Neurosurgery, The First Affiliated Hospital and College of Clinical Medicine of Henan University of Science and Technology, Luoyang, Henan 471003, China

**Keywords:** TRIM family, glioma, epigenetics, immune, genetic alteration

## Abstract

Background: TRIM family molecules have been identified as being involved in the tumor progression of various cancer types. Increasingly, experimental evidence indicates that some of TRIM family molecules are implicated in glioma tumorigenesis. However, the diverse genomic changes, prognostic values and immunological landscapes of TRIM family of molecules have yet to be fully determined in glioma.

Methods: In our study, employing the comprehensive bioinformatics tools, we evaluated the unique functions of 8 TRIM members including TRIM5/17/21/22/24/28/34/47 in gliomas.

Results: The expression levels of 7 TRIM members (TRIM5/21/22/24/28/34/47) were higher in glioma as well as its diverse cancer subtypes than in normal tissues, whereas the expression level of TRIM17 was the opposite, lower in the former than in the latter. In addition, survival analysis revealed that the high expression profiles of TRIM5/21/22/24/28/34/47 were associated with poor overall survival (OS), disease-specific survival (DSS) and progress-free interval (PFI) in glioma patients, whereas TRIM17 displayed adverse outcomes. Moreover, the 8 TRIM molecules expression as well as methylation profiles remarkably correlated with different WHO grades. And genetic alterations, including mutations and copy number alterations (CNAs), in the TRIM family were correlated with longer OS, DSS and progress-free survival (PFS) in glioma patients. Furthermore, through Gene Ontology (GO) and Kyoto Encyclopedia of Genes and Genomes (KEGG) enrichment analysis results of these 8 molecules and their related genes, we found that these molecules may change the immune infiltration of the tumor microenvironment and regulate the expression of immune checkpoint molecules (ICMs), affecting the occurrence and development of gliomas. The correlation analyses between the 8 TRIM molecules and TMB (tumor mutational burden)/MSI (microsatellite instability)/ICMs discovered that as the expression level of TRIM5/21/22/24/28/34/47 increased, the TMB score also increased significantly, while TRIM17 showed an opposite outcome. Further, a 6-gene signature (TRIM 5/17/21/28/34/47) for predicting overall survival (OS) in gliomas was built by using the least absolute shrinkage and selection operator (LASSO) regression, and the survival and time-dependent ROC analyses all were found to perform well in testing and validation cohorts. Results of multivariate COX regression analysis showed that TRIM5/28 are both expected to become independent risk predictors to guide clinical treatment.

Conclusion: In general, the results indicate that TRIM5/17/21/22/24/28/34/47 might exert a crucial influence on gliomas tumorigenesis and might be putative prognostic markers and therapeutic targets for glioma patients.

## INTRODUCTION

Glioma is one of the most common and malignant primary tumors of the central nervous system, composed mainly of glial cells, including astrocytoma, oligodendroglioma, ependyma, anaplastic astrocytoma, glioblastoma, among others [1–3]. Diffuse low-grade and moderate gliomas (WHO Grade II and III) are low-grade subtypes, collectively referred to as low-grade gliomas (LGGs). Patients with LGGs have a longer survival period than patients with high-grade glioma, ranging from 1 to 15 years [4, 5]. Glioblastoma (GBM) is a high-grade subtype, accounting for 54% of gliomas in the United States [6]. The median survival of patients initially diagnosed with GBM was less than 15 months [7, 8], even with optimal surgical resection and subsequent chemoradiotherapy. In general, despite the combined application of surgery, radiotherapy and chemotherapy, the prognosis of glioma patients is difficult to predict. Since many studies have firmly shown that there are interobserver variations in the way of pathological grading diagnosis of gliomas entirely based on histological discrepancies in the past [9–11], the 2016 revision of the WHO Classification of Central Nervous System Tumors introduced molecular parameters, which had been confirmed to be better associated with clinical outcome than histological classification by more and more studies, on the basis of traditional histological classification [5, 12]. Recently, numerous cancer-associated proteins have been identified to play a significant role in the occurrence and development of glioblastoma. Consequently, various inhibitors have been documented to target these crucial proteins in glioma [13, 14]. Therefore, exploring new biomarkers with high specificity and sensitivity and new molecular targets will assist in clarifying the molecular mechanism of gliomas and help to improve the prognosis of patients with gliomas.

Currently, more than 70 TRIM proteins are known, and they are encoded by about 71 genes in humans, some are clustered together [15]. Members of the TRIM family can be defined as ubiquitin E3 ligases because they contain a ring-finger domain. In addition to the RING-finger region, TRIM proteins also have one or two zinc-finger domains, named B-box, and an associated crimp-crimp region. According to the differences in TRIM protein domain, the molecules can be allocated with I~XI subfamilies [16–18].

Members of the TRIM family play an important role in multiple biological processes, including cell proliferation, differentiation, autophagy as well as positively or negatively regulating carcinogenesis [15, 17, 19]. For instance, Feng et al. showed that the loss of TRIM14 leads to the ubiquitination of ZEB2 and the degradation of protein enzymes, leading to the invasion and migration of cancer cells [20]. M. Kikuchi et al. demonstrated that TRIM24 regulates androgen receptors-mediated transcription, thereby negatively regulate cell proliferation and growth in castration-resistant Prostate cancer (CRPC) in collaboration with TIP60 and BRD7, indicating that it promotes CRPC malignancy [21]. Besides, Ji et al. and Zhou et al. clarified the overexpression of TRIM22 and TRIM31 which leads to the proliferation of GBM cells, through regulating the NF-κB signaling pathway [22, 23]. E. J. Horn et al. referred that elevated TRIM32 activates and promotes the carcinogenesis of some experimental skin-related carcinomas by blocking certain stress-induced apoptotic signaling pathways [24]. Recently, increasing number of TRIM proteins are identified to be related to the malignancy and prognosis of cancer [25].

Although the role of TRIM family members in the tumorigenesis and prognosis of several cancers has been partially confirmed [26, 27], these publications represent “just the tip of the iceberg.” Most of the “hidden parts below the water”, represents the role of TRIM family of molecules and their variety of contributions to tumors, especially gliomas. Their impacts in malignant processes are yet to be elucidated. Moreover, the work of the laboratory research is often narrowly focused, researchers cannot always get a glimpse of the whole picture of the iceberg in below water, thus failing to provide continuous and consistent guidance for the research avenues for researchers.

The recent development of bioinformatics disciplines has facilitated this progress, through the integration of data repositories, open access databases, and advanced algorithmic analysis tools, enhancing researchers’ ability to gain profound insights into relevant landscapes.

In summary, we systematically analyzed the expression, prognosis, mutations, and their relationship with cancer grades of different TRIM family members in glioma patients through bioinformatics method and determined that TRIM5, TRIM17, TRIM21, TRIM22, TRIM24, TRIM28, TRIM34 and TRIM47 molecules have a unique role in the development of glioma patients. The above 8 TRIM family molecules maybe play important role in the diagnosis, therapy and prognosis assessment in patients with gliomas in the future.

## MATERIALS AND METHODS

### TRIM family expression pattern in gliomas

The TRIM family mRNA expression profile was investigated by combining the data for normal tissues from the GTEx (http://commonfund.nih.gov/GTEx) database with data from The Cancer Genome Atlas (TCGA) (https://portal.gdc.cancer.gov/) database. Meanwhile, TRIM family mRNA expression levels in different histological subtypes of LGG and GBM were validated using data from GEPIA2 (http://gepia2.cancer-pku.cn/#index) [28], and the data from CGGA (http://www.cgga.org.cn/) [29–33] were applied to analyze the expression and methylation levels of eight TRIM family molecules when altering WHO grades. Furthermore, immunohistochemistry analyses were conducted to observe the distribution and protein level of the TRIM family members in the HPA database (https://www.proteinatlas.org/) [34].

### Prognostic analysis

The connection between the TRIM family expression and the prognosis of patients, including overall survival (OS), disease-specific survival (DSS), and progress-free interval (PFI) in gliomas was examined using Kaplan-Meier curves based on the data from TCGA and CGGA [35]. Cox regression algorithm was applied to the analyses. R survival (version: 3.2-10) and survminer (version: 0.4.9) packages were employed for statistical analysis and visualization.

### Receiver operating characteristic (ROC) analysis

The efficiencies of the TRIM family members prognostic prediction were evaluated by the receiver operating characteristic (ROC) curves using R pROC (version: 1.17.0.1) and ggplot2 (version: 3.3.3) packages [36]. And the gliomas RNAsep data, uniformly processed by the Toil process, were extracted from UCSC Xena (https://xenabrowser.net/datapages/) database [37, 38].

### TCGA data and cBioPortal

cBioPortal is a comprehensive web resource for exploring, visualizing, and analyzing multidimensional cancer genomics data [39, 40]. Brain Lower Grade Glioma (TCGA, PanCancer Atlas) and Glioblastoma Multiforme (TCGA, PanCancer Atlas) datasets with mutations and copy number alterations cases from TCGA, including data from 514, 592 cases with pathology reports respectively, were selected for further analyses of eight TRIM family members. Genetic alterations in TRIM family and their association with overall survival (OS), disease-specific survival (DSS) and progress-free survival (PFI) of glioma patients were displayed as Kaplan-Meier plots and the log-rank test was performed to identify the significance of the diversity between the survival curves. When *P* < 0.05, the difference was considered statically significant.

### Tumor infiltration analysis

The single-sample GSEA (ssGSEA) was performed using the R GSVA package [41] to quantify the correlation between the expression profiles of the TRIM family and the tumor infiltration of 24 immune cell types based on TCGA. Feature gene panels for each immune cell type were obtained from a recent publication [42]. Besides, the Estimation of STromal and Immune cells in MAlignant Tumor (gliomas) tissues using Expression data (ESTIMATE) was employed to explore the immune infiltration landscapes through R estimate (version: 1.0.13) package [43].

### Analysis of the relationship of TRIM family expression and each other as well as TMB/MSI/ICMs

The mRNA-seq data, comprised of tumor mutation burden (TMB) and microsatellite instability (MSI) scores, which was obtained from TCGA [44, 45]. Correlation analyses between the TRIM family expression and each other as well as TMB/MSI, immune checkpoint molecules (ICMs) were performed using Spearman’s method [46–49]. R ggstatsplot and pheatmap packages were applied to analyses and visualizations. *P* value < 0.05 was the significance threshold in this study.

### Enrichment analysis

To understand the potential biological functions of TRIM family and their co-expressed genes, the “Similar Gene Detection” module of GEPIA2 was applied to obtain the top 100 TRIM family members-correlated targeting genes based on the datasets of gliomas, including LGG and GBM. Then R clusterProfiler (version: 3.14.3) and org.Hs.eg.db packages [50] were used to carry out and visualize Gene Ontology (GO) and Kyoto Encyclopedia of Genes and Genomes (KEGG) analyses. *P* < 0.05 and false discovery rate (FDR) <0.05 were considered statistically significant.

### Univariate and multivariate cox regression analysis

Both, univariate and multivariate Cox analysis was used to determine the correlation of TRIM family molecules’ expression and other clinicopathological factors (WHO grade, 1p/19q codeletion, IDH status, gender age, primary therapy outcome) on OS by R survival package based on TCGA data without repeated samples [35, 51]. Factors with *P* value < 0.1 were incorporated in multivariate analysis. *P* < 0.05 was set as the significance threshold for all statistical analyses.

### Development and validation of the TRIM family Gene-Based signature

The entire TCGA gliomas cohort was regarded as a testing cohort and the CGGA gliomas cohort was considered as an external validation cohort. Using the R package “glmnet” [52], we employed the least absolute shrinkage and selection operator (LASSO) analysis regression to narrow down the eight trim family candidates and develop the prognostic model. The optimal tuning parameter (lambda) was determined through tenfold cross-validations. To calculate the risk score, the expression of each gene in the signature was multiplied by its regression coefficient, and then these values were summed. Survival analysis was applied to assess the predictive value of the signature. The “time-ROC” R packages were employed to perform 1-year, 3-year, and 5-year time-dependent receiver operating characteristic (time-dependent ROC) curve analyses.

### Cell culture

In this experiment, human U87 and U251 glioblastoma cell lines were obtained from the Chinese Academy of Medical Sciences (Beijing, China). The cell lines were cultured in high-glucose DMEM complete medium supplemented with 10% fetal bovine serum (FBS) and 1% penicillin/streptomycin. All cells were maintained in a humidified incubator with a 5% CO2 atmosphere at 37°C.

### Transfection

The U87 and U251 glioblastoma cells were transfected with plasmids containing either the trim34-FLAG or FLAG sequences, which were obtained from Miaoling Biotechnology Co., LTD (Jiangsu, China). Alternatively, the cells were transfected with skp2 small interfering RNA (siRNA) using the jetPRIME reagent, following the manufacturer’s guidelines. Control cells were transfected with corresponding empty vectors. The trim5 siRNA sequences utilized were: sense strand, 5′- -3′; and antisense strand, 5′- -3′.

### Cell viability assay

Cell viability was detected by CCK-8 assay. The cells cultured above were seeded into 96-well culture plates (4 × 10^3^ cells/well). Following treatment, 10 μl of CCK-8 reagent was added to each well. Plates were cultured continuously for 2 hours at 37°C with 95% air and 5% CO2. The absorbance values at 450 nm were detected with a microplate reader. Data were shown as the survival rate relative to the blank control.

### Colony formation assay

Colony formation assays were performed on U87 and U251 cell lines to assess clonogenic capacity. Cells were seeded at a density of 500 cells per well in 6 cm dish culture plates and allowed to adhere for 24 hours at 37°C with 5% CO2. Following the treatments, cells were incubated for 14 days, with media changes every three days.

At the end of the incubation, colonies were fixed with a methanol-acetic acid solution (3:1, v/v) and stained with 0.5% crystal violet. Colonies, defined as clusters of at least 50 cells, were counted manually or using a colony counter.

### Transwell assay

The migratory and invasive capacities of U87 and U251 cell lines were assessed utilizing Transwell assays in chambers featuring 8-μm pore size membranes (Corning, USA). Cells were introduced into the upper chambers at a density of 2 × 10^4^ cells/well in 200 μL of DMEM, supplemented with 1% FBS. Concurrently, the lower chambers were filled with 600 μL of DMEM containing 20% FBS to establish a chemotactic gradient. To evaluate the invasive potential, the upper chamber membranes were pre-coated with 10 μL of Matrigel (BD Biosciences, USA), creating a simulated extracellular matrix barrier. After a 24-hour incubation period, non-migratory or non-invasive cells remaining on the upper membrane surface were carefully removed using a cotton swab. The cells that had successfully traversed the membrane to the lower surface were fixed with ethanol and subsequently stained with 0.2% crystal violet. To quantify the migrated and invaded cells, five random fields were selected and examined at 100x magnification. This experiment was carried out in triplicate to confirm the consistency and reliability of the obtained results.

### Statistical analysis

In the *in vitro* studies, the data displayed represent the mean ± standard error of the mean derived from three independent experiments and were analyzed using SPSS 20.0 software. Comparisons between two groups were performed using the independent *t*-test, whereas the one-way analysis of variance was employed for comparing multiple groups. A *p*-value of less than 0.05 was deemed to indicate statistical significance.

### Data availability

The datasets generated and/or analysed during the current study are available in the UCSC Xena repository (https://tcga.xenahubs.net). Data used included the Cancer Genome Atlas (TCGA, http://cancergenome.nih.gov/), the Genotype-Tissue Expression projects (GTEx, https://commonfund.nih.gov/GTEx), the Chinese Glioma Genome Atlas database (CGGA, http://www.cgga.org.cn/). And some analyses and visualization of this study are derived from public databases, as follows. Gene Expression Profiling Interactive analysis 2 database (GEPIA2, http://gepia2.cancer-pku.cn/#index), cBioPortal database (https://www.cbioportal.org/).

## RESULTS

### Aberrant expression of TRIM family in gliomas

**Figure 1:**
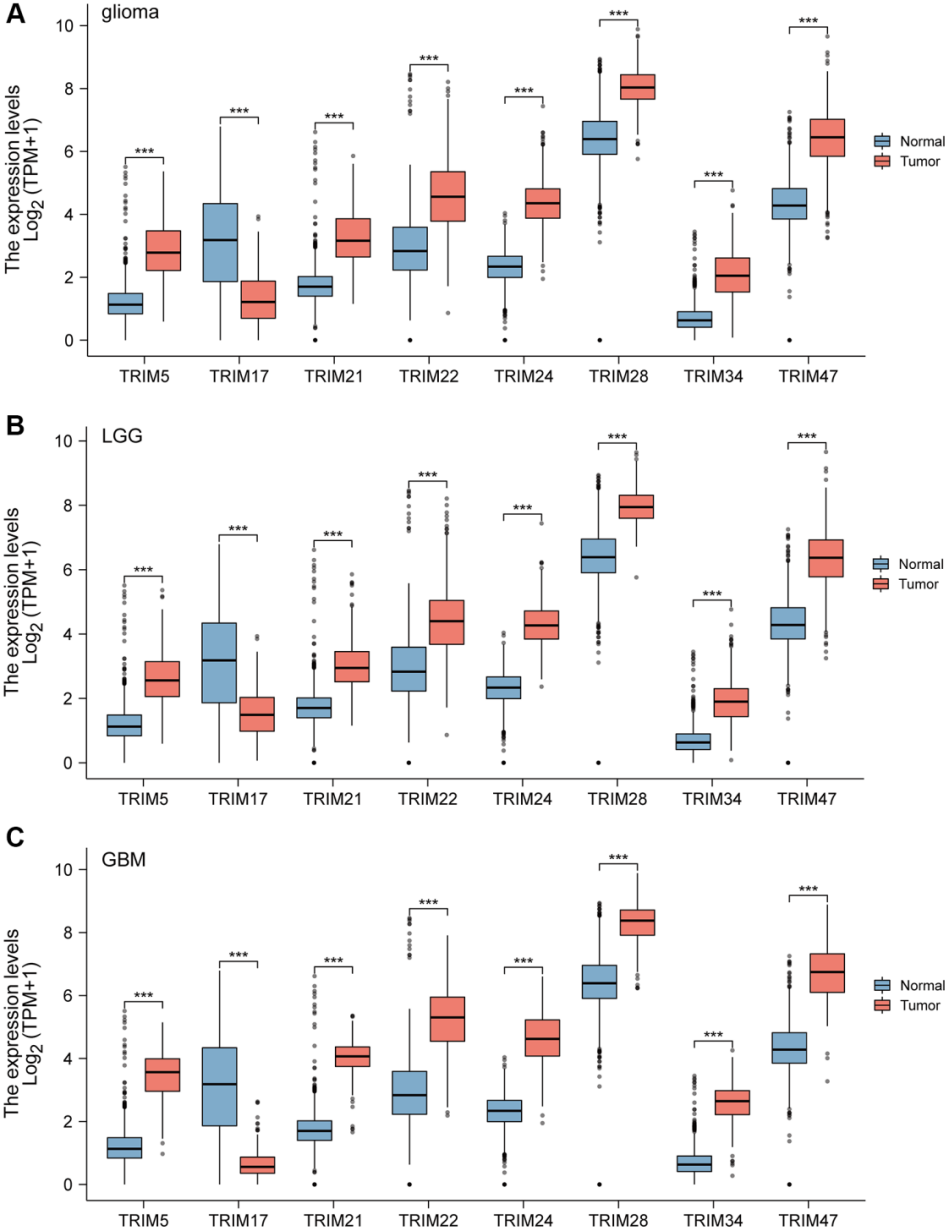
**The mRNA expression of diverse Tripartitemotif (TRIM) 5/17/21/22/24/28/34/47 in glioma tissues and normal tissues.** mRNA expressions of TRIM5/21/22/24/28/34/47 are found to be over-expressed in glioma, LGG, and GBM tissues compared to normal samples. Whereas, the expression level of TRIM17 is lower in the glioma, LGG, and GBM tissues than in normal tissues (**A**–**C**). ns, *p* ≥ 0.05; ^*^p < 0.05; ^**^p < 0.01; ^***^p < 0.001.

**Figure 2:**
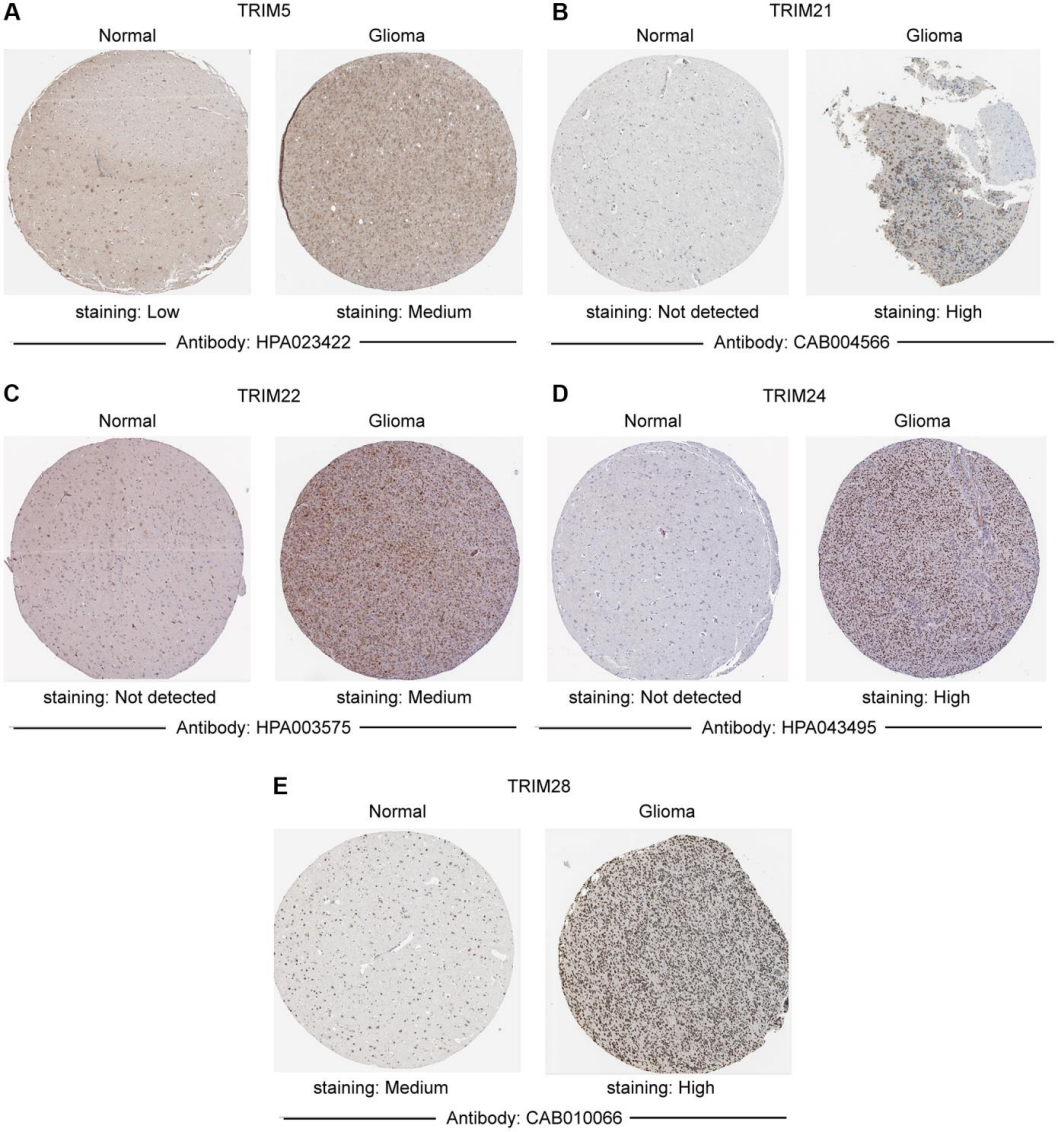
**Immunohistochemistry staining of representative.** (**A**) TRIM5; (**B**) TRIM21; (**C**) TRIM22; (**D**) TRIM24; (**E**) TRIM28 molecules based on the Human Protein Atlas.

**Figure 3:**
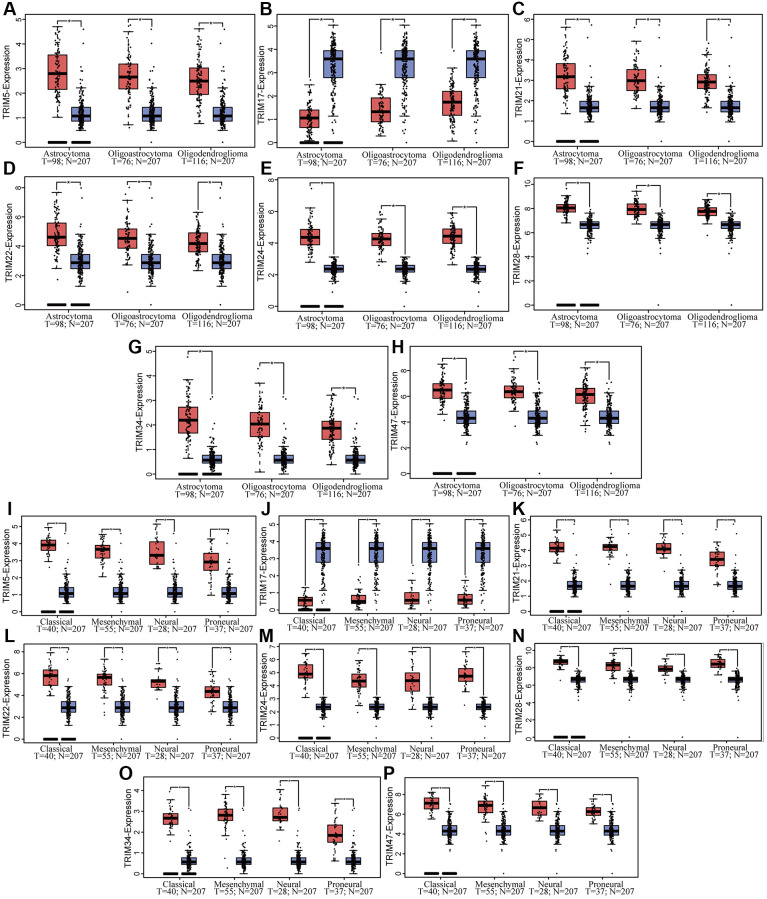
**Correlation between TRIM family mRNA expression and different glioma tissue subtypes in patients.** The mRNA expressions of TRIM family across LGG and GBM tissue subtypes (**A**–**H**), while mRNA expression of TRIM family across different glioma subtypes (**I**–**P**).

Combining normal tissue data from the GTEx database with the related data from TCGA, we evaluated the mRNA expression profile of the TRIM family and discovered that TRIM5/21/22/24/28/34/47 were overexpressed in glioma, LGG and GBM tissues. While TRIM17 performs in an adverse state, showing a lower expression level in glioma, LGG, and GBM tissues compared to the normal (Figure 1). In addition, immunohistochemical analysis was applied to observe the distribution and protein levels of TRIM family members. As shown in Figure 2, TRIM 5/21/22/24/28 exhibited more elevated expression levels in gliomas. At the same time, through GEPIA2, we discovered that the mRNA expression of TRIM5/21/22/24/28/34/47 was significantly up-regulated in different LGG and GBM histological subtypes (Figure 3A, 3C–3I, 3K–3P), while the expression of TRIM17 was down-expressed in diverse gliomas subtypes (Figure 3B, 3J).

**Figure 4:**
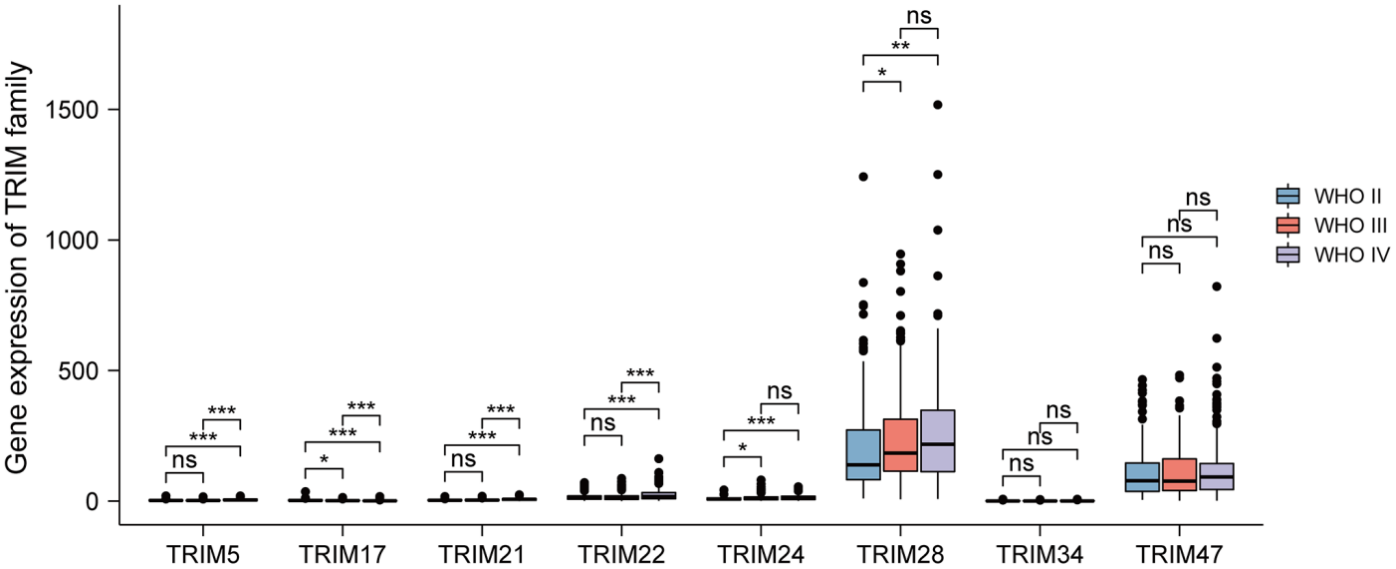
**Relationship between diverse TRIM family expression level and different WHO grades.** ns, *p* ≥ 0.05; ^*^p < 0.05; ^**^p < 0.01; ^***^p < 0.001.

To further probe the relation between the expression levels of the 8 TRIM family molecules and different WHO classifications (WHO I-IV), based on the data of CGGA, a correlation analysis was performed. The results showed that TRIM24/28 significantly up-expressed in WHO III compared with WHO II, while TRIM17 performed adverse character in this comparison. TRIM5/21/22/24/28 significantly up-expressed in WHO IV compared with WHO II, while TRIM17 exhibited adverse features; TRIM5/21/22 significantly up-expressed in WHO IV compared with WHO III, while TRIM17 showed opposite character (Figure 4).

### Prognostic significance of TRIM family

**Figure 5:**
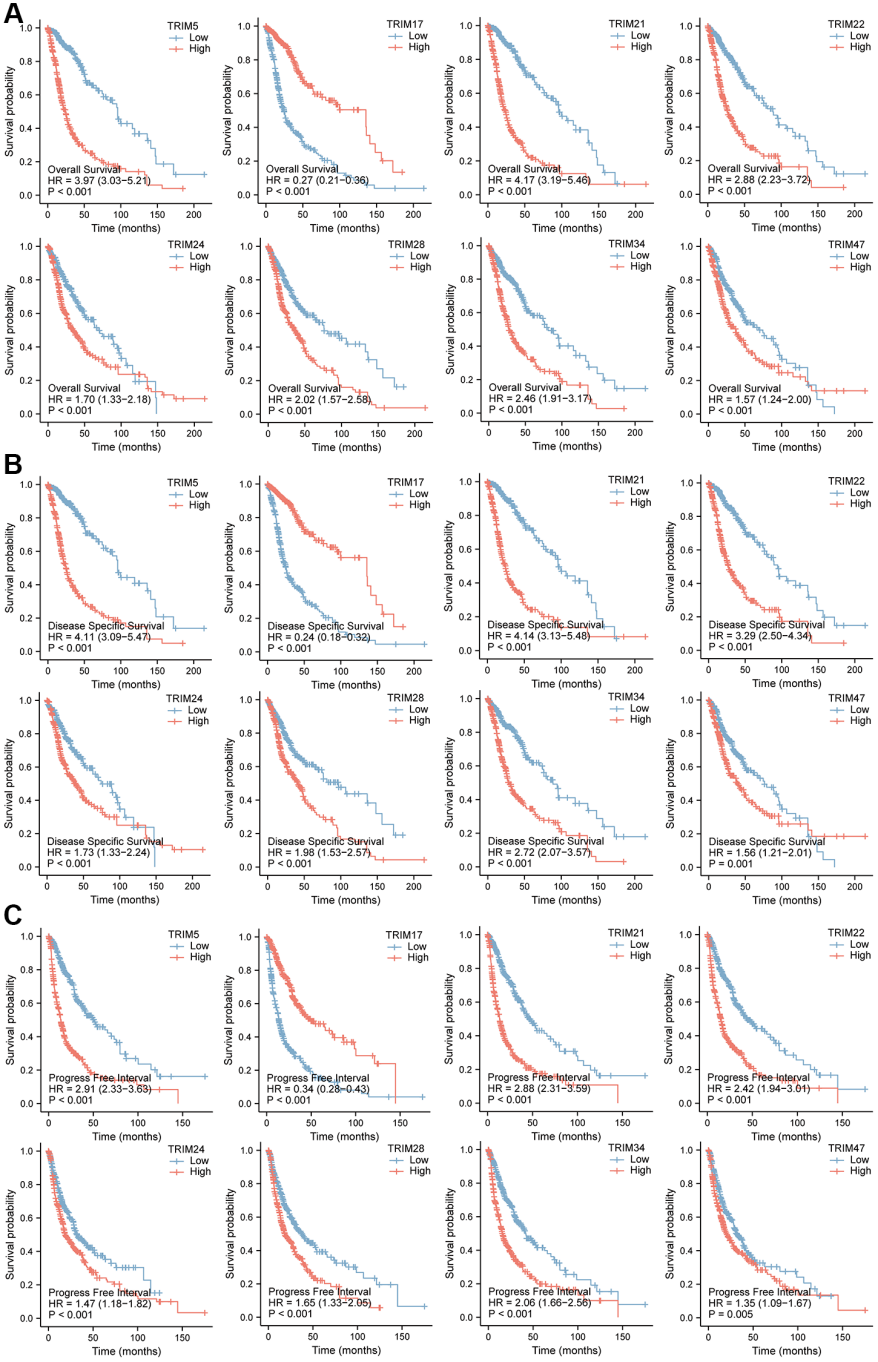
**Prognostic feature of mRNA expression of distinct TRIM family members in glioma patients.** The OS, DSS, and PFI survival curves comparing patients with high and low TRIM family member expression in gliomas are shown (**A**–**C**). Abbreviations: OS: overall survival; DSS: disease-specific survival; PFI: progress-free interval.

The relationship between TRIM family expression and patient prognosis through Kaplan-Meier curves were examined based on TCGA data, to obtain the overall survival (OS), disease-specific survival (DSS), and progression-free interval (PFI) profiles of glioma patients (Figure 5). Among them, patients with high expression profiles of TRIM5/21/22/24/28/34/47 had shorter OS, DSS and PFI than patients with low corresponding molecules expression (*P* < 0.001), while patients with high TRIM17 expression displayed opposite outcomes, showing longer OS (*P* < 0.001), DSS (*P* < 0.001) and PFI (*P* = 0.005) than the adverse corresponding patients.

**Figure 6:**
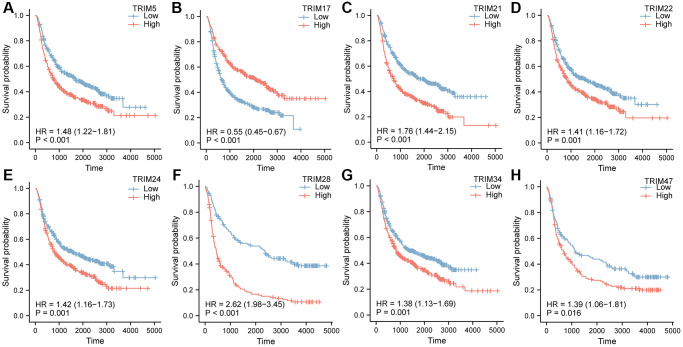
OS survival curves of glioma patients stratified by the expression level of TRIM family through CGGA database (**A**–**H**).

Simultaneously, the OS survival data of patients with glioma stratified by the expression level of the TRIM family, obtained in the CGGA database, was applied to analysis (Figure 6). TRIM5/21/22/24/28/34/47 exhibited remarkedly similar results in TCGA. Among them, patients with high expression of TRIM5/21/22/28/34/47 had a shorter survival probability than those with low expression of corresponding molecules, while high TRIM17 expression tended to attain longer survival probability.

### Receiver operating characteristic (ROC) analysis

**Figure 7:**
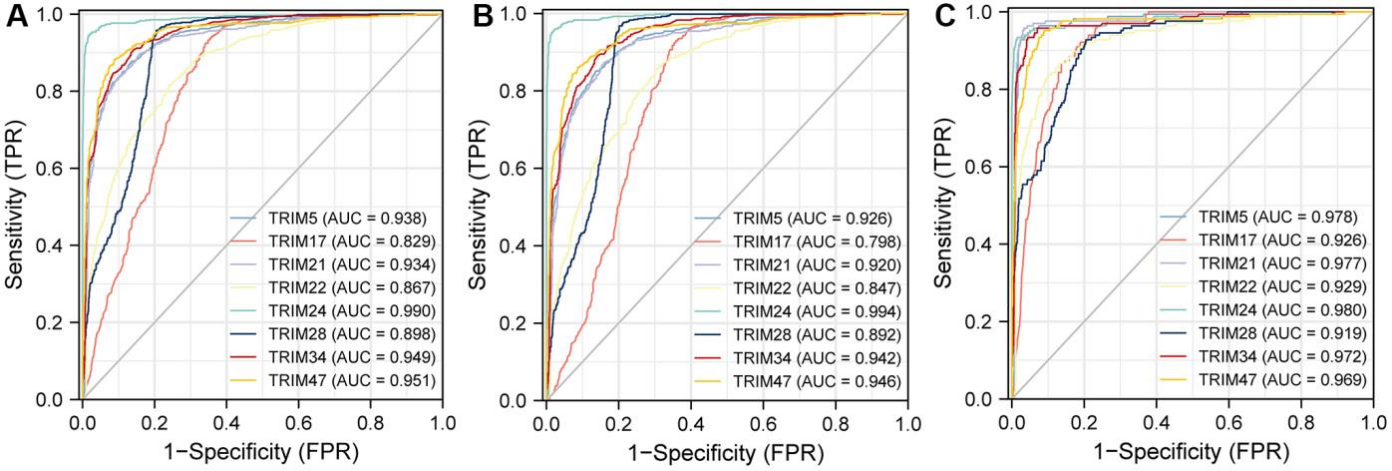
The area under the curve values for receiver operator characteristic (ROC) curves for TRIM family members across gliomas (**A**), LGG (**B**) and GBM (**C**).

To better understand the efficiencies of the TRIM family members prognostic prediction, we evaluated ROC curve analysis (Figure 7). Results demonstrated that TRIM5(AUC = 0.938)/17(AUC = 0.829)/21(AUC = 0.934)/22(AUC = 0.867)/24(AUC = 0.990)/28(AUC = 0.898)/34(AUC = 0.949)/TRIM47(AUC = 0.951) exposed excellent accuracy in gliomas. And TRIM5(AUC = 0.926)/17(AUC = 0.798)/21(AUC = 0.920)/22(AUC = 0.847)/24(AUC = 0.994)/28(AUC = 0.892)/34(AUC = 0.942)/TRIM47(AUC = 0.946) has acceptable accuracy in LGG. TRIM5(AUC = 0.978)/17(AUC = 0.926)/21(AUC = 0.977)/22(AUC = 0.929)/24(AUC = 0.980)/28(AUC = 0.919)/34(AUC = 0.972)/TRIM47(AUC = 0.969) displayed outstanding accuracy in GBM.

### Epigenetic alterations analysis

**Figure 8:**
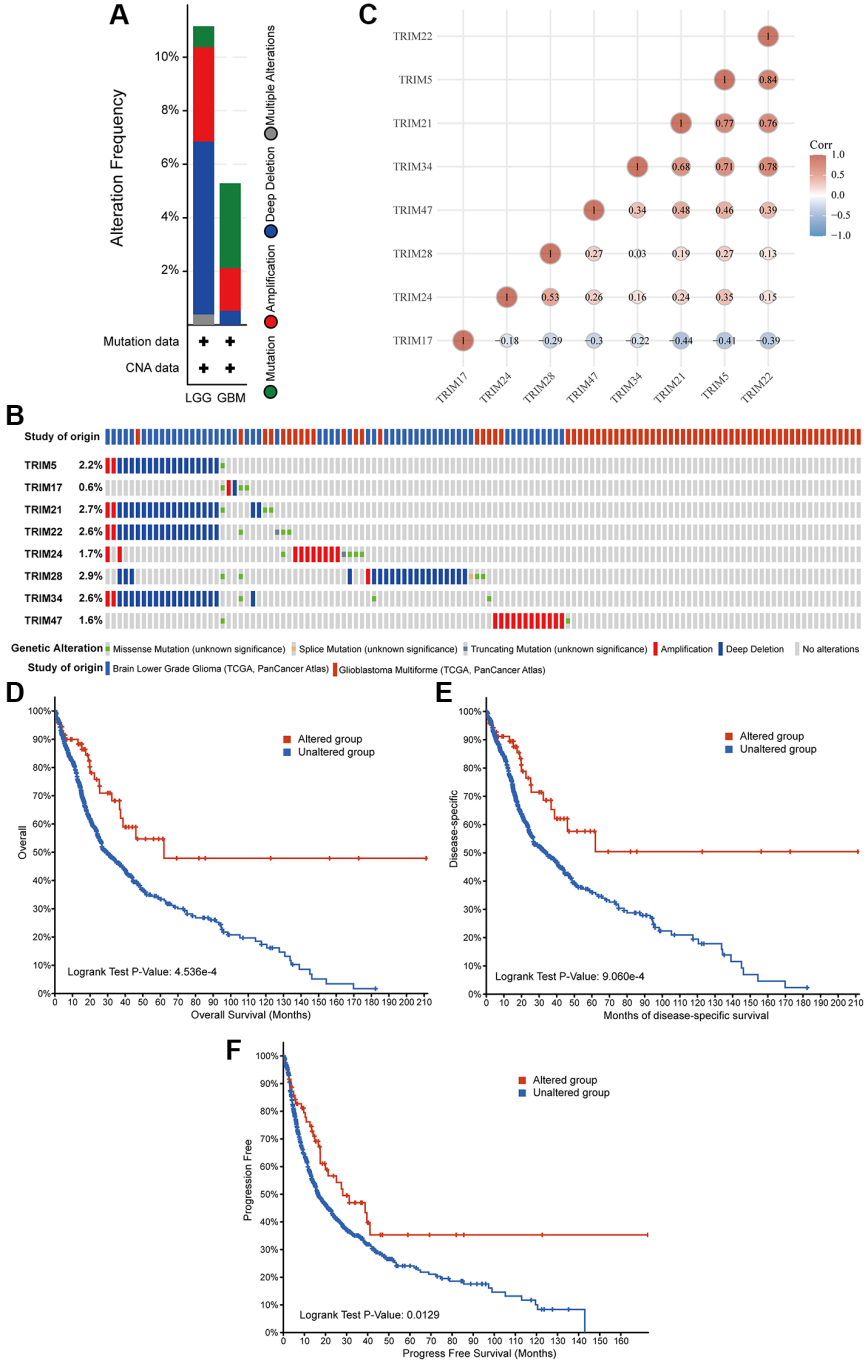
**Genetic alterations in eight TRIM family members and their association with prognosis of glioma patients.** Summary of alterations in different expressed TRIM families in gliomas (**A**, **B**). Correlations of different TRIM family members with each other (**C**). Genetic alterations in TRIM family were correlated to longer OS (**D**), DSS (**E**), PFS (**F**) of glioma patients. Abbreviation: PFS: progress-free survival.

**Figure 9:**
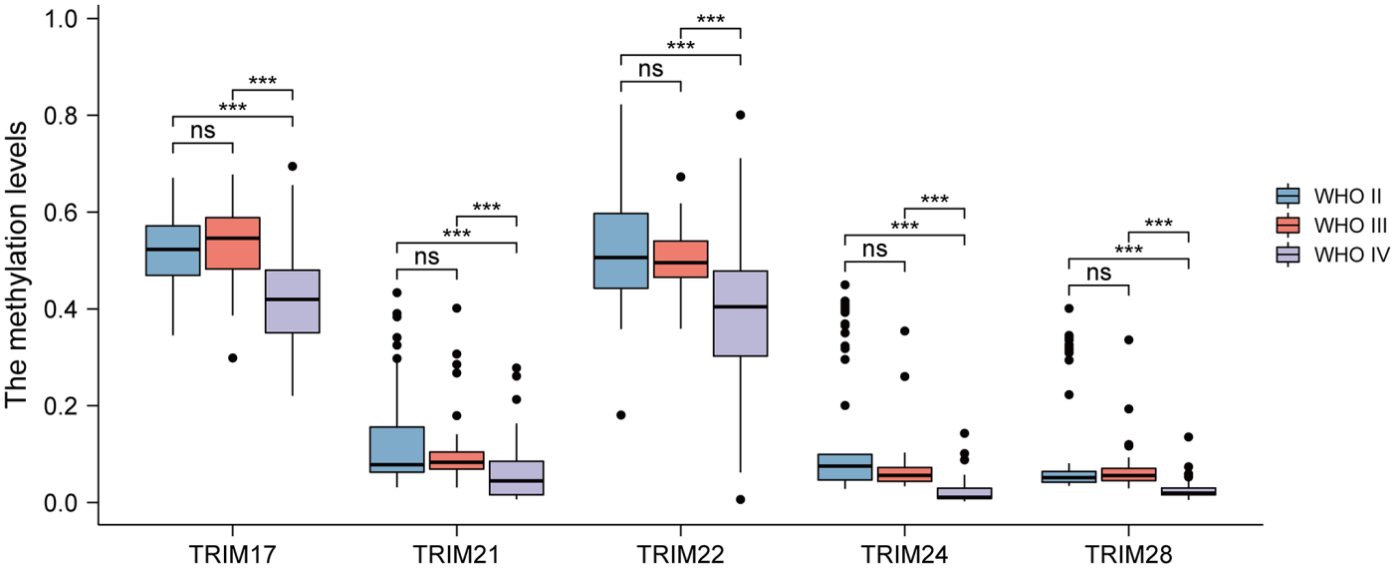
**Analyses of the methylation level of the TRIM family in different WHO grades of gliomas.** ns, *p* ≥ 0.05; ^*^p < 0.05; ^**^p < 0.01; ^***^p < 0.001.

Epigenetic alteration plays a vital role in early malignancies. The TRIM family alterations, including mutations and copy number aberrations, and correlations were analyzed by using the cBioPortal online tool for LGG and GBM. The genetic alterations of TRIM family were varied, 11.15% of 511 patients with LGG and 5.29% of 378 patients with GBM respectively (Figure 8A). The specific genetic changes of the TRIM family molecules and their alteration rates are shown in Figure 8B, respectively. In addition, to tease out the correlations between the 8 TRIM molecules, analyzing their mRNA expression via Spearman correlation analysis for gliomas was conducted. The consequences exposed the noteworthy relationship between TRIM17 and 7 other TRIM molecules which all show negative correlations. The correlations of other family molecules are shown (Figure 8C). Furthermore, we analyzed the relationship of genetic alterations in the TRIM family with OS, DSS and progress-free survival (PFS) of glioma patients. Results from the Kaplan-Meier plot and log-rank test uncovered that, genetic alterations in TRIM family were related to longer OS (*P* = 4.536E-4), DSS (*P* = 9.060E-3) and PFS (*P* = 0.0129) of glioma patients (Figure 8D–8F). These observed discoveries that the genetic changes of TRIM family may crucially affect the prognosis of glioma patients. Besides, extracting the data from CGGA, we performed methylation analyses, the outcomes showed that the methylation levels of TRIM17/21/22/24/28 in WHO IV gliomas were significantly lower than that in WHO II and WHO IV (*P* < 0.001) (Figure 9).

### Tumor infiltration analysis

**Figure 10:**
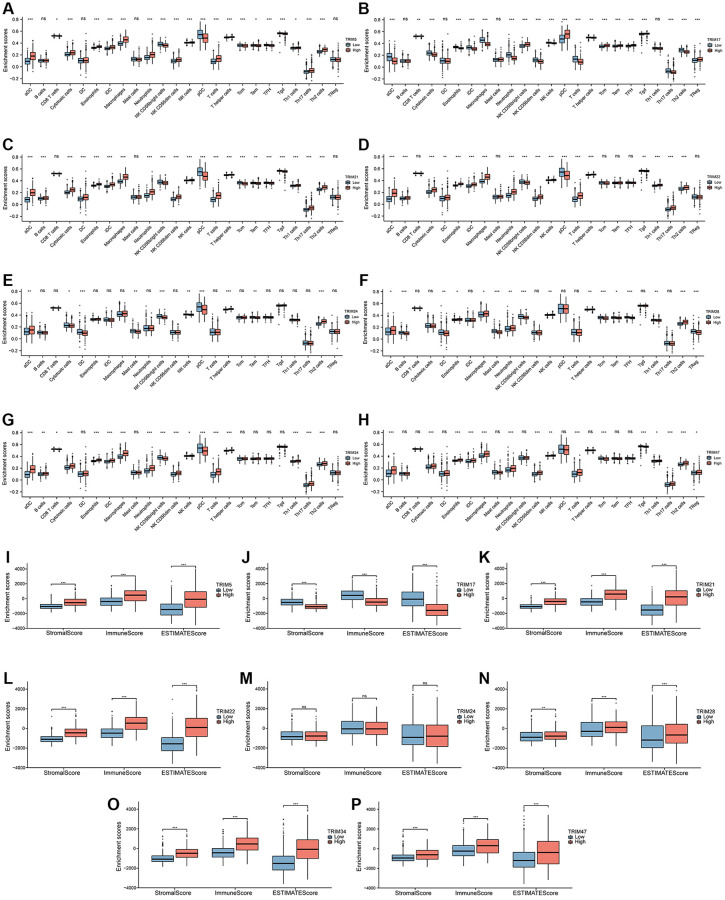
**Immune infiltration landscapes of TRIM molecular family in gliomas.** Correlation between TRIM family members' expression and 24 tumor-infiltrating immune cell types (**A**–**H**). Distribution of stromal score, immune score and ESTIMATE score in high- versus low-TRIM family expression groups (**I**–**P**). ns, *p* ≥ 0.05; ^*^p < 0.05; ^**^p < 0.01; ^***^p < 0.001.

The correlation between the expression of TRIM family members and 24 tumor-infiltrating immune cell types (Figure 10A–10H) was quantified based on TCGA. The results showed that the expression of TRIM5 and activated DCs (aDCs), Cytotoxic cells, Eosinophils, immature DCs (iDCs), Macrophages, Neutrophils, NK CD56^dim^ cells, NK cells, T cells, T helper cells, Th17 Cells, Th2 cells (*P* < 0.001) and Th1 cells (*P* < 0.05) have a significant positive correlation; while with NK CD56^bright^ cells, plasmacytoid DCs (pDCs), T central memory (Tcm) cells, T follicular helper (TFH) cells, T gamma delta (Tgd) cells (*P* < 0.001), CD8 T cells and T effector memory (Tem) cells (*P* < 0.05) have a significant negative correlation. The expression of TRIM17 and NK CD56^bright^ cells, pDCs, Tcm, Tem, TFH, Tgd, Treg (*P* < 0.001) and CD8 T cells (*P* < 0.01) have a significant positive correlation; with aDCs, Cytotoxic cells, Eosinophils, iDCs, Macrophages, Neutrophils, NK CD56^dim^ cells, NK cells, T cells, Th17 cells, Th2 cells (*P* < 0.001) and T helper cells (*P* < 0.01) have a significant negative correlation. The expression of TRIM21 and aDCs, Cytotoxic cells, DCs, Eosinophils, iDCs, Macrophages, Neutrophils, NK CD56^dim^ cells, NK cells, T cells, T helper cells, Th1 cells, Th17 cells, Th2 cells (*P* < 0.001) and B cells (*P* < 0.01) have a significant positive correlation; with NK CD56^bright^ cells, pDCs, Tcm, TFH, Tgd (*P* < 0.001) and Tem (*P* < 0.01) have a significant negative correlation. The expression of TRIM22 is significantly positively correlated with aDCs, B cells, Cytotoxic cells, Eosinophils, iDCs, Macrophages, Neutrophils, NK CD56^dim^ cells, NK cells, T cells, T helper cells, Th1 cells, Th17 cells, Th2 cells (*P* < 0.001) and DC (*P* < 0.01); while it significantly negatively related with NK CD56^bright^ cells, pDCs (*P* < 0.001) and Tgd (*P* < 0.01). The expression of TRIM24 is significantly positively correlated with T helper cells, Th2 cells (*P* < 0.001), and aDCs (*P* < 0.01); while DCs, NK CD56^bright^ cells, pDCs (*P* < 0.001), Mast cells, NK cells, Tem (*P* < 0.01) and Cytotoxic cells (*P* < 0.05) have a significant negative correlation. The expression of TRIM28 is significantly positively correlated with Th2 cells (*P* < 0.001), Neutrophils, NK cells, T helper cells (*P* < 0.01), aDCs, Eosinophils and Macrophages (*P* < 0.05); while with B cells, Mast cells, NK CD56^bright^ cells, Tcm, TFH (*P* < 0.001), DC, Th1 cells, Treg (*P* < 0.01) and Tem (*P* = 0.05) have a significant negative correlation. The expression of TRIM34 was significantly positively correlated with aDCs, Cytotoxic cells, Eosinophils, iDCs, Macrophages, Neutrophils, NK CD56^dim^ cells, T cells, T helper cells, Th1 cells, Th17 cells, Th2 cells (*P* < 0.001), B cells (*P* < 0.01) and NK cells (*P* < 0.05). And it has a significant negative correlation with NK CD56^bright^ cells, pDCs (*P* < 0.001), and CD8 T cells (*P* < 0.05). The expression of TRIM47 was significantly positively correlated with aDCs, Cytotoxic cells, Eosinophils, iDCs, Macrophages, Neutrophils, NK CD56^dim^ cells, T cells, Th17 cells, Th2 cells (*P* < 0.001), NK cells (*P* < 0.01) and Treg (*P* < 0.05); while Tcm and Tgd (*P* < 0.001) have a significant negative correlation. While immune cells with no significant difference are indicated in the Figure 10A–10H.

To infer tumor purity and stromal and immune cell admixture in gliomas (Figure 10I–10P), ESTIMATE algorithm was employed to attain the outcomes, which exhibited that the stromal, immune, and ESTIMATE scores of the matrix of the expression group with high expression of TRIM5/21/22/28/34/47 are higher than those with low expression of TRIM5/21/22/28/34/47, while the three scores of the expression group with high expression of TRIM17 are lower than those with low expression of TRIM17, showing an opposite trend again. However, TRIM24 did not show a significant difference in this analysis.

### Correlation between the expression of TRIM family molecules and TMB/MSI, ICMs in gliomas

**Figure 11:**
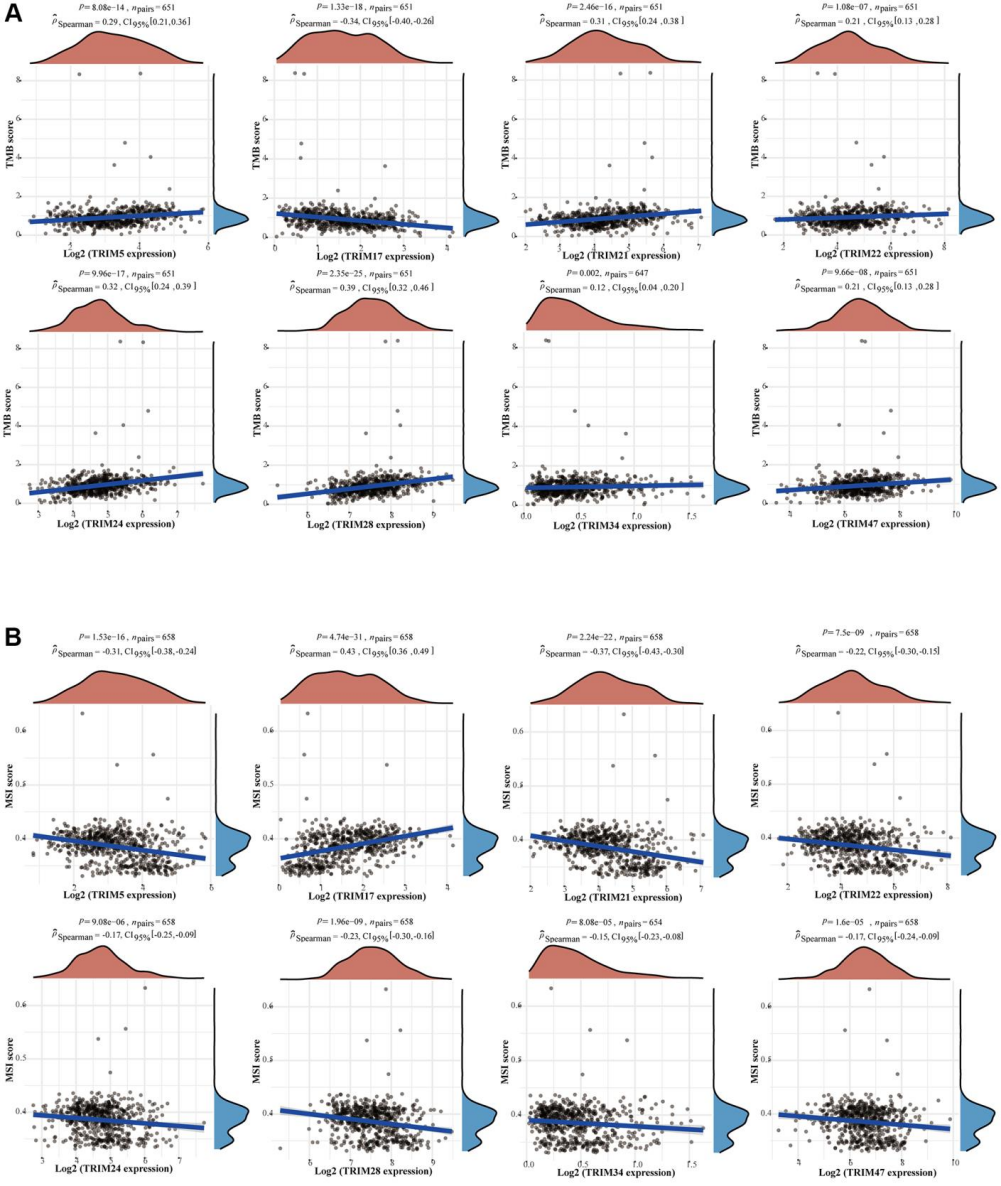
**Correlation between the expression profiles of TRIM family members and TMB/MSI in gliomas.** Relationship between TRIM family expression and TMB (**A**) or MSI (**B**). The horizontal axis in the figure represents the expression distribution of the genes, and the ordinate is the expression distribution of the TMB/MSI scores. The density curve on the right represents the distribution trend of the TMB/MSI score; the upper-density curve represents the distribution trend of the gene. Abbreviations: TMB: tumor mutational burden; MSI: microsatellite instability.

**Figure 12:**
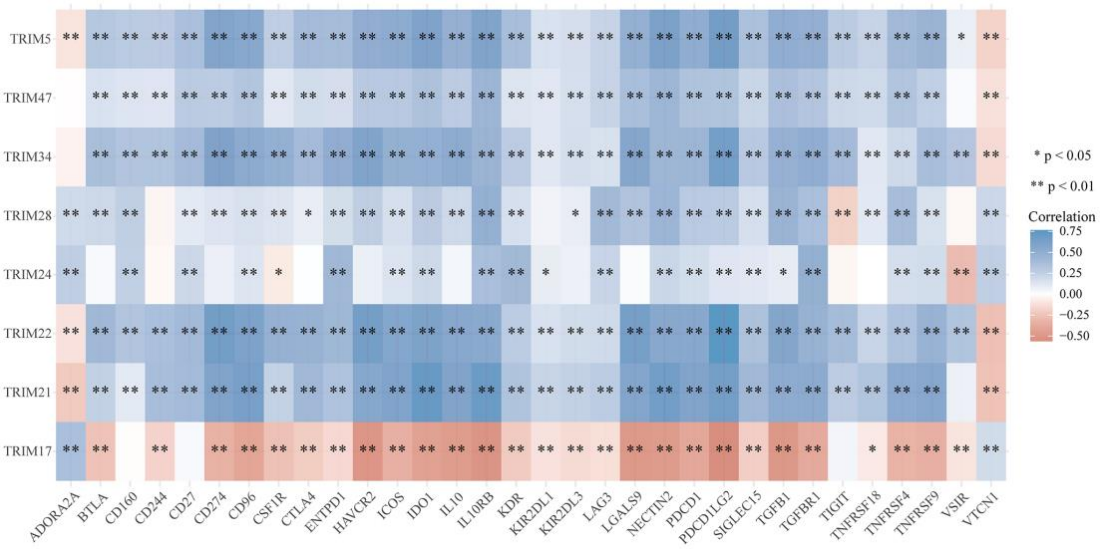
**Correlation analyses of the expression profiles of TRIM family with immune checkpoint molecules in gliomas.** The horizontal and vertical ordinates represent genes, and different colors represent correlation coefficients (in the diagram, blue represents positive correlation, red represents negative correlation), and the darker the color represents the two stronger correlations. ^*^p < 0.05; ^**^p < 0.01; ^***^p < 0.001.

To study the correlation between TRIM family expression profiles and tumor mutation burden (TMB) and microsatellite instability (MSI) in gliomas, we discovered that TRIM5/21/22/24/28/34/47 were remarkably positively correlated with TMB (*P* < 0.05), and TRIM17 was significantly negatively correlated with TMB (*P* < 0.05). Besides, TRIM5/21/22/24/28/34/47 was significantly negatively correlated with MSI (*P* < 0.05), while TRIM17 was remarkably positively correlated with MSI (*P* < 0.05) (Figure 11) through detecting the relationship between TRIM family expression and TMB/MSI. In addition, the outcomes of the relationship between TRIM family molecules expression and ICMs were shown in Figure 12.

### Putative functional role of TRIM family

**Figure 13:**
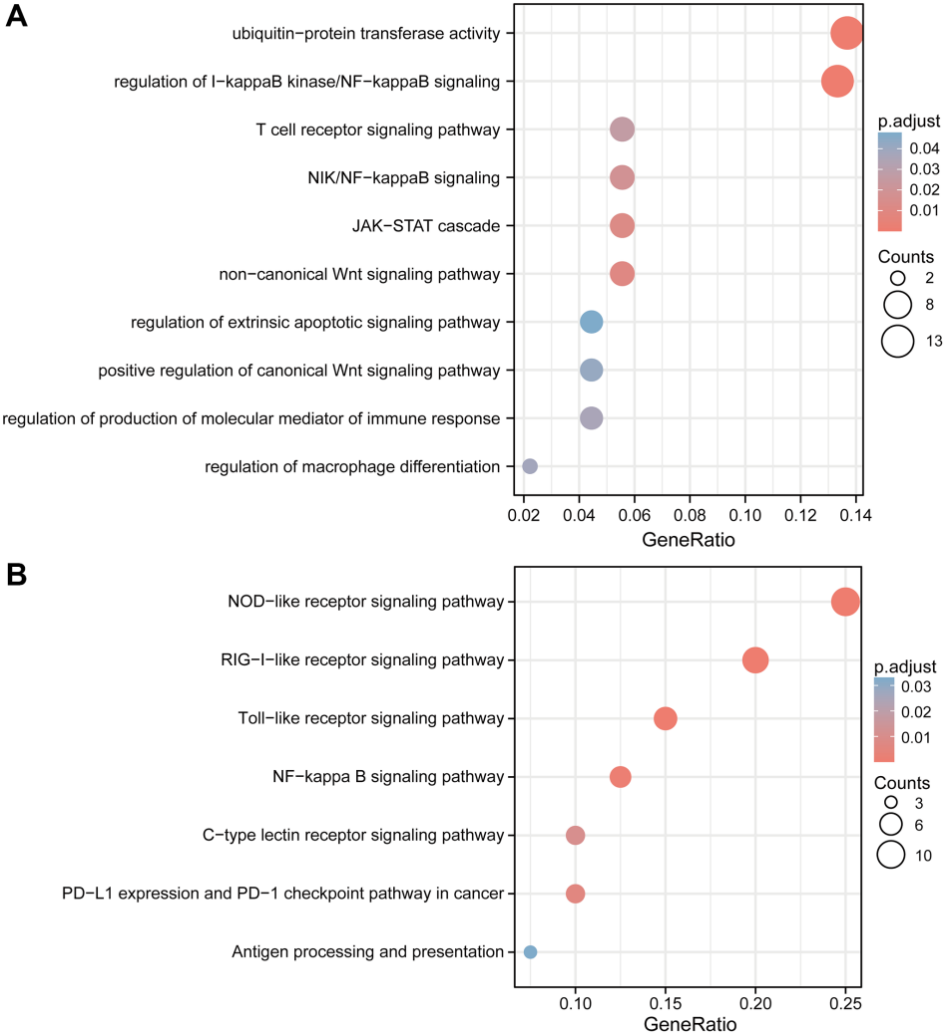
**GO and KEGG enrichment analyses of TRIM family members and their co-expression genes in gliomas.** Bubble charts of GO (**A**) and KEGG (**B**) terms. Abbreviations: GO: Gene Ontologies; KEGG: Kyoto Encyclopedia of Genes and Genomes.

The remarkably enriched GO terms are composed of ubiquitin-protein transferase activity, regulation of I-kappaB kinase/NF-kappaB signaling, T cell receptor signaling pathway, NIK/NF-kappaB signaling, JAK-STAT cascade, non-canonical Wnt signaling pathway, regulation of extrinsic apoptotic signaling pathway, positive regulation of canonical Wnt signaling pathway, regulation of production of molecular mediator of immune response and regulation of macrophage differentiation (Figure 13A). The KEGG pathway related to NOD-like receptor signaling pathway, RIG-I-like receptor signaling pathway, Toll-like receptor signaling pathway, NF-kappa B signaling pathway, C-type lectin receptor signaling pathway, PD-L1 expression, and PD-1 checkpoint pathway in cancer and Antigen processing and presentation were also significantly enriched (Figure 13B).

### Cox regression analysis

Univariate Cox regression analysis showed that all eight TRIM family molecules were significantly associated with the OS (*P* < 0.001). In addition, the multivariate Cox regression analysis demonstrated that TRIM5 (HR 2.005, 95% CI = 1.180–3.408, *p* = 0.010) and TRIM28 (HR 1.749, 95% CI = 1.122–2.728, *p* = 0.014) were independent risk factors for OS. The results are summarized in Table 1.

**Table 1:** Univariate and Multivariate Cox analysis of TRIM family and other clinical-pathological factors for OS.

**Characteristics**	**Total (*N*)**	**Univariate analysis**		**Multivariate analysis**	
		**Hazard ratio (95% CI)**	***P* value**	**Hazard ratio (95% CI)**	***P* value**
**Gender**	698		0.071		
Female	297	Reference		Reference	
Male	401	1.250 (0.979–1.595)	0.073	1.577 (1.004–2.477)	**0.048**
**Age**	698		**<0.001**		
≤60	555	Reference		Reference	
>60	143	4.696 (3.620–6.093)	**<0.001**	3.976 (2.363–6.691)	**<0.001**
**WHO grade**	636		**<0.001**		
G2 and G3	468	Reference		Reference	
G4	168	9.538 (7.243–12.560)	**<0.001**	3.142 (1.053–9.373)	**0.040**
**IDH status**	688		**<0.001**		
WT	246	Reference		Reference	
Mut	442	0.116 (0.089–0.151)	**<0.001**	0.413 (0.252–0.676)	**<0.001**
**Primary therapy outcome**	464		**<0.001**		
PD and SD	260	Reference		Reference	
PR and CR	204	0.205 (0.117–0.359)	**<0.001**	0.286 (0.153–0.533)	**<0.001**
**TRIM5**	698		**<0.001**		
Low	348	Reference		Reference	
High	350	3.938 (3.010–5.153)	**<0.001**	2.005 (1.180–3.408)	**0.010**
**TRIM17**	698		**<0.001**		
Low	349	Reference		Reference	
High	349	0.279 (0.213–0.366)	**<0.001**	0.669 (0.438–1.022)	0.063
**TRIM21**	698		**<0.001**		
Low	348	Reference		Reference	
High	350	4.424 (3.372–5.804)	**<0.001**	1.279 (0.796–2.054)	0.309
**TRIM22**	698		**<0.001**		
Low	348	Reference		Reference	
High	350	2.811 (2.180–3.624)	**<0.001**	0.976 (0.618–1.540)	0.917
**TRIM24**	698		**<0.001**		
Low	349	Reference		Reference	
High	349	1.700 (1.328–2.177)	**<0.001**	0.694 (0.423–1.137)	0.147
**TRIM28**	698		**<0.001**		
Low	348	Reference		Reference	
High	350	2.021 (1.577–2.590)	**<0.001**	1.749 (1.122–2.728)	**0.014**
**TRIM34**	698		**<0.001**		
Low	347	Reference		Reference	
High	351	2.212 (1.729–2.830)	**<0.001**	1.168 (0.724–1.883)	0.525
**TRIM47**	698		**<0.001**		
Low	348	Reference		Reference	
High	350	1.593 (1.252–2.028)	**<0.001**	0.995 (0.648–1.528)	0.981

### Identification of a 6 gene signature in the TCGA cohort and validation of the risk signature

**Figure 14:**
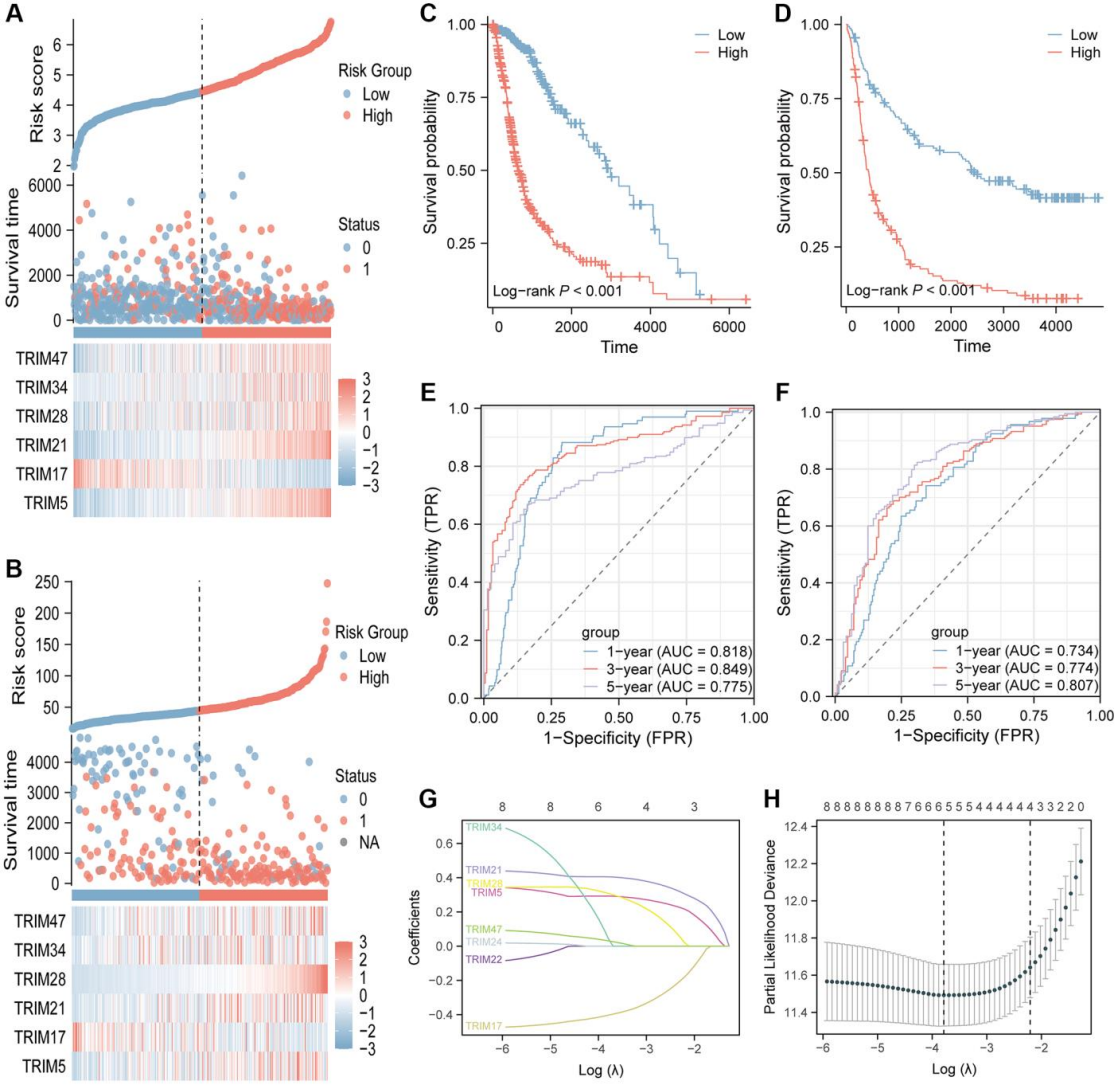
**Identification and validation of the 6-gene risk signature.** The risk score distribution, the survival status and overall survival time of patients and the expression of the six TRIM genes in testing (**A**) and validation (**B**) cohorts. Kaplan-Meier survival analysis in testing (**C**) and validation (**D**) cohorts suggested that high-risk group had poor OS than low risk group. Time-dependent ROC curves of the gene signature for predicting 1-year, 2-year, and 3-year overall survival in testing (**E**) and validation (**F**) cohorts. LASSO regression of the eight TRIM candidates (**G**). Cross-validation for tuning the parameter selection in the LASSO regression (**H**). LASSO, Least absolute shrinkage and selection operator.

By performing LASSO regression analysis, a 6-gene signature (TRIM 5/17/21/28/34/47) was constructed according to the optimum λ value (Figure 14G, 14H). Patients from the TCGA datasets were stratified into low and high-risk groups based on the median. A notable difference in OS was detected between the low- and high-risk groups (*P* < 0.001, Figure 14C). Time-dependent ROC analysis was applied to evaluate the sensitivity and specificity of the prognostic model, and the AUC was separately 0.818 for 1-year survival, 0.849 for 3-year survival, and 0.775 for 5-year survival (Figure 14E). In addition, 325 patients from the CGGA datasets were divided into low- and high-risk groups. OS of the low-risk group is also better than that of the high-risk group (*P* < 0.001, Figure 14D). The AUC was separately 0.734 for 1-year survival, 0.774 for 3-year survival, and 0.807 for 5-year survival (Figure 14F). Besides, the risk score distribution, the survival status and overall survival time of patients in testing and validation cohorts, as well as the expression of the 6 TRIM genes in high- and low-risk groups were presented in Figure 14A, 14B.

### Knockdown of trim5 or trim34 attenuated the proliferation, invasion, and migration capabilities of glioma cells

**Figure 15:**
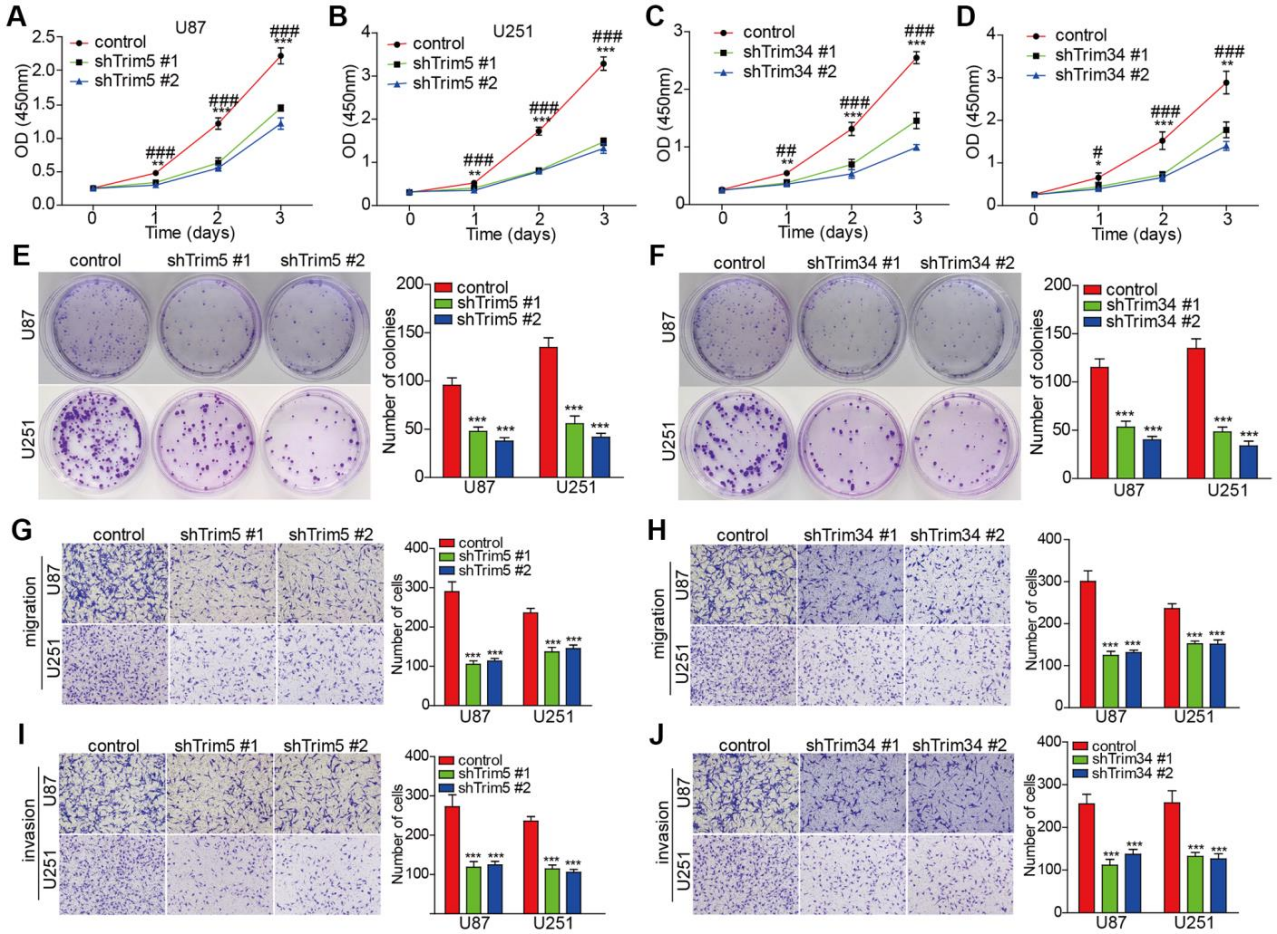
**Knockdown of trim5 or trim34 attenuated the proliferation, invasion, and migration capabilities of glioma cells.** (**A**–**D**) Cell proliferation was evaluated in U87 and U251 cells with or without Trim5 or Trim34 knockdown by CCK-8 assay, Trim5 or Trim34 shRNA #1 versus control shRNA, ^*^P < 0.05, ^**^P < 0.01, ^***^P < 0.001; Trim5 or Trim34 shRNA #2 versus control shRNA; ^#^*P* < 0.05, ^##^*P* < 0.01, ^###^*P* < 0.001. (**E**, **F**) Colony formation capability was examined in U87 and U251 cells with or without Trim5 or Trim34 knockdown by colony-formation assay, Trim5 or Trim34 shRNA versus control shRNA, ^*^P < 0.05, ^**^P < 0.01, ^***^P < 0.001 (**G**–**J**) Cell migration and invasion ability was evaluated in U87 and U251 cells with or without Trim5 or Trim34 knockdown by transwell assay, Trim5 or Trim34 shRNA versus control shRNA, ^*^p < 0.05, ^**^p < 0.01, ^***^p < 0.001.

To validate the functional roles of the TRIM family in glioma, we conducted further *in vitro* experiments. However, since other TRIM molecules mentioned in our study, except for TRIM34 and TRIM5, have already been validated in glioma *in vitro* experiments from previous studies [23, 53–55], we focused our investigations on TRIM34 and TRIM5. As shown in the Figure 15, knockdown of trim5 or trim34 attenuated the proliferation, invasion, and migration capabilities in U87 and U251 cells, suggesting that TRIM34 and TRIM5 function as oncogenes in glioma. These findings are consistent with the results obtained through bioinformatics analysis.

## DISCUSSION

In this study, we screened out 8 molecules in our self-designed indicators from the TRIM family of molecules in gliomas for the differential expression, prognostic value, and other aspects. And the present research is the first time to explore the mRNA and protein expression as well as prognostic values of different TRIM family members in gliomas. Our findings will contribute to available knowledge, improve treatment designs, and enhance the accuracy of prognosis for patients with gliomas.

Until now, almost no one has deeply explored the image of TRIM5 molecules in the occurrence, development, and malignancy of gliomas. Lesseur et al. had reported that oral and pharyngeal cancers combined were associated with loci at TRIM5, implying TRIM5’s putative function in tumorigenesis and development [56]. Results of differential analysis in our research revealed that the mRNA and protein expression of TRIM5 was higher in gliomas than in normal tissues. Moreover, TRIM5 expression also correlated with the clinical characteristics, including WHO grades as well as histological subtypes of the patients with glioma. To evaluate the prognostic value of TRIM5 in glioma patients, we discovered that the higher TRIM5 expressed, the poorer OS, DSS, PFI they would be. And since *p* < 0.05 in the multivariate cox regression analysis of OS, TRIM5 is noteworthily expected to become an independent risk predictor. Xiao et al. had demonstrated that TRIM17 overexpression significantly inhibited cell proliferation in their CCK-8 and colony formation assays [57]. However, there are only a few explorations on the characteristics of TRIM17 in gliomas. Our study disclosed that the expression of TRIM17 was lower in glioma and its subtype samples than in normal ones. More importantly, higher mRNA expression of TRIM17 was also significantly related to a longer OS, DSS, PFI of glioma patients. TRIM21 overexpression is an oncogenic event in many types of cancers, including glioma, breast cancer and others [58, 59]. Research demonstrates that by regulating cell proliferation, migration, and senescence, TRIM21 overexpression promotes glioma progression. This, is demonstrated in our report, the mRNA and protein expression of TRIM21 was much higher in gliomas. Moreover, he increased expression of TRIM21 was significant, positively correlated with the highest tumor grade and diverse subtypes in gliomas. Consistent with the role as an oncogene, TRIM21 overexpression, was also significantly correlated with poor OS, DSS, PFI in all of the patients with glioma. TRIM22 is highly expressed in several tumors, including glioblastoma and colon cancer [23, 60]. For instance, Liu et al. discovered that Linc01207 promotes colon cancer cell proliferation and invasion by regulating miR-3125/TRIM22 axis. In this report, the expression of TRIM22 in gliomas was higher than that in normal tissues in mRNA and protein levels. Results, also indicated that the TRIM22 expression profile was positively correlated with histological subtypes in patients with glioma and was significantly up-regulated in GBM. Zhang et al. clarified that activating the PI3K/Akt signaling pathway, TRIM24 promoted glioma progression and enhanced chemoresistance. But the prognostic role of TRIM24 in glioma has yet to be investigated. Here, we demonstrated that TRIM24 had higher expression levels at both the RNA and protein levels in glioma samples, and its expression was correlated with tumor histological subtypes in gliomas. Higher TRIM24 expression was correlated with poor OS, DSS, PFI, in all of the patients with glioma with significance. TRIM28, a transcriptional co-factor, targets many genes with pleiotropic biological activities [24]. Qi et al. had reported that down-regulating TRIM28 increased p21 expression and induced cell cycle of glioma cells to arrest at the G1 phase, thereby exerted a critical influence on glioma progression, all evidence indicating TRIM28 role as an oncogenic contributor in glioma carcinogenesis [61]. In our report, we demonstrated that the mRNA and protein expression profiles of TRIM28 were higher in glioma tissues than in normal tissues, with, expression partially correlated with tumor grades in patients with glioma. Moreover, high TRIM28 expression was significantly correlated with poor OS, DSS, PFI in glioma patients, which seemed consistent with its role of TRIM28 as a tumor activator. In addition, TRIM28 shows expectations of becoming an independent risk factor, as revealed through our multivariate analysis. To date, the expression and role of TRIM34 in gliomas was poorly reported, using a similar approach; through differential analysis, we revealed that TRIM34’s expression in glioma samples and their different histopathological subtypes are significantly up-regulated. Moreover, high TRIM34 expression significantly correlated with poor OS, DSS, PFI. TRIM47 has a role in promoting the development of glioma by ubiquitination and degradation of FOXO1 [62]. In addition, the study had indicated that knockdown of TRIM47 inhibited cell proliferation, as well as cell migration and invasion through the inactivation of Wnt/β-catenin pathway [55]. In this study, we found that the up-expression of TRIM47 in glioma and its histological subtypes tissues, were similar to the findings by Wei et al. and Chen et al. High TRIM47 mRNA expression led to the reduced OS, DSS and PFI of glioma patients-a significant observation.

ROC curve has been widely used due to its outstanding function in the assessment of the performance of a diagnostic test [63]. However, almost no research has focused on exploring the diagnostic performance of the above 8 TRIM molecules in gliomas. Therefore, our analyses emerged as the times require. In the diagnostic test evaluation of glioma, all the AUC values surpassed 0.8, reaching excellent levels. In the diagnostic performance of GBM, the AUC values of 8 molecules are all above 0.9, also reaching outstanding levels. Our results strongly indicated that the increased expression of all 8 TRIM molecules in glioma tissues might play an important role in glioma, suggesting that these molecules may be potential diagnostic indicators and therapeutic targets for glioma patients and it is very promising for early screening of glioma patients in the future.

The genetic mutations and copy number changes of somatic cells have been revealed to be closely related to the occurrence and development of tumors, including gliomas. The best explanation is that diffuse astrocytic and oligodendroglial tumors are classified by the presence of isocitrate dehydrogenase 1 or 2 (IDH1/2) mutation in the 2016 World Health Organization (WHO) classification of central nervous system (CNS) tumors for the impact of specific gene mutations on the progression and patient outcome of glioma [64]. In addition, Zhao et al. found that after the copy number of KIF23 alterations, its expression level is increased, which in turn leads to tumorigenesis and the development of gliomas [65]. Our experimental results showed that the TRIM family molecules are genetically altered in gliomas. Although the frequency of genetic alterations is not as high as expected, it is sufficient to have a significant better influence on prognosis.

DNA methylation is a major form of epigenetic modification of DNA that regulates the gene expression without altering the sequence of DNA. Hypermethylation within promoter regions often leads to the silencing or inactivation of tumor suppressor genes in cancerous cells [66, 67]. Our results demonstrated that DNA methylation of TRIM family members, specifically, TRIM17/21/22/24/28, perform significantly lower methylation levels collectively, in association with gliomas of WHO IV versus WHO II and III. While the WHO II and III gliomas did not show sharply significant differences. Interestingly, these observations indicates that the epigenetic changes in the methylation level may play potential role in the progression of glioma from LGG to GBM. The relationship between DNA methylation of 8 TRIM family members reported in this research would benefit from further in-depth studies.

Furthermore, TRIM family members and their co-expression genes in gliomas through GO and KEGG pathway analysis were functionally annotated, and these results elucidated the regulation of I-kappaB kinase/NF-kappaB signaling, NIK/NF-kappaB signaling, JAK-STAT cascade, non-canonical Wnt signaling pathway and positive regulation of canonical Wnt signaling pathway in GO as well as NOD-like receptor signaling pathway and NF-kappa B signaling pathway in KEGG. A large number of earlier research had shown that these pathways played a pivotal role in the malignant process in gliomas [68, 69]. Inferring from these results, the TRIM molecules tested in this paper, are very likely to perform, as yet unknown functions, and in conjunction with Chen et al. studies, verifying the influence of TRIM47 on the oncogenesis of glioma cells via the Wnt/β-catenin pathway [55], the role of TRIM molecules with tumor research is promising.

Tumor cells can interact with immune microenvironment and influence the occurrence and development of tumor, and the immune infiltrating cells in tumor are a critical part of immune microenvironment [70, 71]. Hence, we estimated the immune microenvironment of the 8 TRIM family molecules. Our results clearly indicate that the 8 TRIM molecules are inextricably linked with various immune cells, the specific landscapes shown in Figure 10A–10H. With the exception of TRIM24, whose ssGSEA performance in the analysis of estimates was unsatisfactory, all other TRIM molecules show an encouraging result. Specifically, the high expression groups of TRIM5/21/22/28/34/47 had higher immune scores, indicating that these molecules may promote the malignant progression of glioma by regulating the function of immune infiltrating cells. However, the high expression of TRIM17 tended to obtain a lower stromal score, immune score, and ESTIMATE score, which was predicted with the results explored before when determining its prognostic value.

Immunotherapy has transformed the clinical oncology landscape, in recent years, contributing to significant improvements in long-term survival in some cancer patients. Some molecules can affect the immunotherapeutic effect of glioma patients by affecting on immune checkpoint molecules [72, 73]. In previous study, it has been reported that there are some limitations in CNS disease immunotherapies and glioma immunotherapies are largely unavailable. Recently, more and more investigation exploring the glioma immunotherapies and cumulating evidence has been reported to be useful. In current study, our immune checkpoint correlation analysis showed that, with the exception of TRIM17, which has a negative correlation with PD-1 (PDCD1), the remaining 7 TRIM molecules all have a positive correlation with PD-1. TRIM24 seems an outlier, has a frustrating performance among the analyses, since no significant correlation between it with many immune checkpoint molecules, such as BTLA, CD274, CTLA4, and others are evident. TRIM5/21/22/28/34/47 molecules generally showed positive correlations with ICMs, further verifying our idea/intuition that these molecules promote the malignancy of gliomas by inhibiting the function of immune white cells. What is consistent with our original understanding is that TRIM17 is negatively correlated with most of the ICMs included in the analysis. These indicated that these molecules are likely to shine on the stage of immune targeted therapy in the future.

Among the investigated biomarkers in immune checkpoint targeted therapy to date, tumor mutational burden (TMB) has recently emerged as a potential predictor of response to immunotherapy in various tumor types [74, 75]. Tumors with high levels of TMB are thought to express more cancer-specific antigens that may sensitize them to immunotherapy [75–78]. In addition, research had demonstrated that higher TMB was related to worse prognosis, older age, higher grade, and higher immune checkpoint expression [79]. Wang et al. proved the above observations, through the study of a large number of samples, that TMB is associated with poor outcomes in diffuse glioma [80]. The expression levels of TRIM family members correlated strongly with TMB in gliomas. TRIM5/21/22/24/28/34/47 exhibited positive correlations, while TRIM17 exhibited negative correlations, which indicated that patients with gliomas with high expression of TRIM5/21/22/24/28/34/47 or low expression of TRIM17 may benefit from emerging immunotherapy, despite the implications for poorer prognosis.

Whereas the exploration of MSI is not as promising result seems diametrically opposite to TMB, where TRIM5/21/22/24/28/34/47 exhibited negative correlations, and TRIM17 exhibited a positive correlation. However, McCord et al. suggested that not all cells in a hypermutated glioma may actually be DNA mismatch repair-deficient, a very crucial factor that causes high instability of microsatellites [81, 82]. Moreover, the observation of 624 glioma samples, by Eckert et al. that mismatch repair deficiency does not play a major role in the pathogenesis of glial neoplasms [83]. Research by D. A. Lundin et al. clarified that microsatellite length alterations are infrequent and the frequency of MSI is low in sporadic adult gliomas [84]. These indicate the status of MSI in determining options to use immunotherapy for glioma patients may not be as important as TMB.

In summary, identifying patients among the glioma population who are likely to have good outcomes with immunotherapy may be complemented due to the correlation results of TMB as well as MSI. However, further research would allow for examining the contradictions.

To further deepen the understanding of the TRIM molecular family, we constructed a 6-gene prognostic signature based on the expression profile of eight TRIM candidates and the corresponding clinical information, which was then found to perform well in the TCGA and CGGA datasets, further indicating the crucial role these TRIM members exert on gliomas tumorigenesis.

No research process can be perfectly or completely designed to resolve biologically complex processes associated with tumor cells, our research has identified many limitations, exposing a pathway for future research. On one hand, all the data analyzed in our study were obtained from different online databases, which may be responsible for background heterogeneity, further studies with larger sample sizes are required to confirm our findings. On the other hand, this research, essentially a desktop study, exploited the vast data available mainly via bioinformatics platforms, analysis of these data may be significant pointers for experimental testing and verification. Consequently, further *in vivo* experiments should allow for these “predictive” results to be examined and may provide deeper understanding and some desirable results.

## CONCLUSIONS

In general, through the use of databases and datasets such as TCGA, we have conducted comprehensive explorations and found a lot of compelling results on the differential expression status and prognosis of molecules of the 8 TRIM family molecules, as well as their correlation with clinical factors and diagnostic value, etc. At the same time, we also conducted a multi-omics, multi-angle, and multi-functional analysis of the genetic changes of these molecules, including mutations, CNAs, and methylation. What’s more, our immune analysis results explained the unique immune landscape of these eight molecules. In addition, we conducted additional *in vitro* experiments to validate the results obtained through bioinformatics analysis. However, this experiment only stays at the stage of insufficient rudimentary theoretical verification, and the actual more objective and in-depth mechanism needs to be exploded by researchers.
